# Hasn’t Child Abuse Been Overlooked? An Evaluation of Abused Children Who Visited the Emergency Department with Sentinel Injuries

**DOI:** 10.3390/children11111389

**Published:** 2024-11-15

**Authors:** Han Bit Kim, Hyun Noh

**Affiliations:** Department of Emergency Medicine, Soonchunhyang University Bucheon Hospital, Bucheon 14584, Republic of Korea; 109149@schmc.ac.kr

**Keywords:** child abuse, sentinel injury, infants, emergency department

## Abstract

Objective: Effective child abuse intervention requires understanding its prevalence. While obtaining a comprehensive national estimate of child abuse cases is challenging, sentinel injuries—minor yet unusual injuries like bruises or wounds in pre-cruising-age children—can provide an indicative measure. Using the National Emergency Department Information System (NEDIS) data, this study aimed to gauge the prevalence of sentinel injuries using diagnostic codes in children under 12 months who visited emergency centers in South Korea and to evaluate the extent of child abuse screening in these cases. Methods: This cross-sectional study used diagnostic codes indicative of sentinel injuries previously defined using the Delphi method. This study, using NEDIS data, included children under 12 months who visited emergency centers nationwide from 2014 to 2021 for reasons of injury. Children injured in car accidents were excluded. Independent variables included patient demographics, the injury mechanism, intentionality, the route of arrival, the emergency center level, the triage level, and specialist consultation. Dependent variables were the presence of a sentinel injury code, and whether diagnostic tests for child abuse were conducted. Results: Based on NEDIS and national statistical data, the frequency from 2014 to 2021 averaged 2501 per 100,000 of the population. Of the 186,065 patients studied, 63,131 (33.9%) had a diagnostic code corresponding to a sentinel injury. The proportion of patients undergoing diagnostic tests for suspected child abuse was 36.9% for those with sentinel injuries and 43.8% for those with other codes. The percentage of children with sentinel injuries receiving diagnostic tests increased annually, from 32.4% in 2014 to 54.4% in 2021. By institution, the rates were 36.4% for regional emergency centers, 38.4% for local emergency centers, and 20.0% for local emergency institutions. Conclusions: A significant proportion of children presenting to emergency departments had sentinel injury codes. However, the rate of diagnostic tests conducted for suspected child abuse in these cases remains suboptimal. Although there has been an upward trend in testing rates in recent years, there is a pressing need for increased attention to and enhancement in screening for child abuse among children with sentinel injury codes.

## 1. Introduction

Child abuse is a global health issue that has immediate and long-term effects on the physical and psychological well-being of children [1]. Despite its significance, accurately estimating the prevalence of child abuse is challenging due to underreporting and differences in identification practices of child abuse [2,3]. In South Korea, following the enactment of the Special Act on the Punishment, etc., of Child Abuse Crimes in 2014, public attention towards child abuse has increased. Consequently, reported cases of child abuse rose from about 10,000 in 2014 to 52,000 in 2021 [4]. However, these figures represent only reported cases, and the extent of unreported child abuse remains difficult to ascertain. Accurate statistical data are crucial for responding to and preventing child abuse, but developing comprehensive national statistics on child abuse has been a challenge [5,6].

In South Korea, the child abuse detection rate is 3.6 per 1000 children, lower than rates reported in the United States (up to 22 per 1000) and Australia (8 per 1000), with medical professionals making up only 1.0% of child abuse reports [7,8,9]. Furthermore, recent studies indicate that approximately 29–32% of adults report experiencing abuse in childhood, suggesting a substantial rate of undetected child abuse cases [10].

The authors of this study aimed to assess the scale of child abuse nationwide by determining the prevalence of children with sentinel injuries visiting emergency centers nationwide. Sentinel injuries are specific forms of injury that may appear prior to severe child abuse incidents. To do this, the study utilized national emergency center data, which do not precisely record sentinel injuries, nor is it easy to verify them individually. Therefore, the authors operationalized diagnostic codes considered indicative of sentinel injuries through a Delphi method developed by child abuse experts in previous research [11].

A prior study found that 27.5% of children with substantiated abuse had previously experienced a sentinel injury, underscoring the importance of focusing on sentinel injury cases to identify unreported abuse [12]. Since emergency departments can serve as critical windows of opportunity for identifying child abuse, this study aims to estimate the prevalence of sentinel injuries among emergency department visits and determine whether diagnostic evaluations for child abuse screening were conducted based on the type of injury and emergency center level, using operational definitions from prior research.

## 2. Methods

### 2.1. Study Design and Setting

This study was designed as a retrospective cross-sectional study, focusing on pediatric patients who visited emergency centers nationwide in South Korea. The study protocol was approved by the Soonchunhyang University Hospital institutional review board (IRB file no. 2023-02-005-001). The data for the study were obtained from the National Emergency Department Information System (NEDIS). The NEDIS is compiled by the National Emergency Medical Center (NEMC) under South Korea’s Ministry of Health and Welfare. The NEDIS is a real-time data collection and information system created to gather both clinical and administrative details from emergency medical centers across the country. Established in 2003, the NEDIS plays a crucial role in evaluating the quality of emergency medical services and serves as a foundational resource for research and policy development in emergency medicine. More comprehensive information on the NEDIS database structure and variables can be found in other studies [13,14]. Utilizing NEDIS data, this study included children under 12 months of age who visited emergency centers nationwide from 2014 to 2021 due to injuries. Only patients with trauma were included in the study, and children injured in car accidents were excluded. To determine the incidence of sentinel injuries per 100,000 of the population, the number of children under 12 months of age was obtained using the National Statistics Portal.

### 2.2. Operational Definition of Sentinel Injuries

In a prior study by the authors, a Delphi study was conducted by eight experts in pediatric emergency medicine who specialized in child abuse [11]. To prepare for the study, a preliminary search was conducted on PubMed and Google Scholar using the keywords “sentinel injury” and “child abuse”. The potential sentinel injuries were categorized into four groups: bruises, burns, open wounds, and human bites or subconjunctival hemorrhage. For each diagnosis, the survey queried whether it could be suspected as a sentinel injury based on its anatomical location (scalp/face/neck, trunk/thorax/buttock, genitourinary organ, upper arm, lower arm, thigh, lower leg) and the child’s age (6 months, 9 months, 12 months). Each round of the survey was accompanied by a video conference, and the survey was conducted using Google Forms and Excel sheets. A diagnosis was defined as a sentinel injury if it was agreed upon by at least five out of the eight experts.

### 2.3. Variables

The independent variables in this study included patient demographics, the mechanism of injury, intentionality, the route of arrival, the level of the emergency center, the triage level, and specialist consultation. The dependent variables were the presence of a sentinel injury code and whether any diagnostic tests for child abuse screening were conducted, including a skeletal survey and any X-ray survey. For the purposes of this study, conducting a diagnostic test was defined as performing at least one of the following: a skull X-ray, chest X-ray, humerus X-ray, femur X-ray, brain imaging (brain CT or MRI), AST/ALT, or a fundus exam. A skeletal survey was defined as having performed all of the following: a skull X-ray, chest X-ray, humerus X-ray, and femur X-ray. Finally, any X-ray survey was defined as having performed at least one of the following: a skull X-ray, chest X-ray, humerus X-ray, or femur X-ray.

### 2.4. Association Between Diagnostic Test and Type of Sentinel Injury

The study categorized types of sentinel injuries as bruises, burns, open wounds, and human bites or subconjunctival hemorrhage. The types of diagnostic tests were divided into categories: any X-ray survey, brain imaging, AST/ALT, and a fundus exam. The study compared the differences in the execution of these diagnostic tests according to the type of sentinel injury.

### 2.5. Association Between the Diagnostic Test and the Level of the Emergency Center

The study conducted a subgroup analysis of children who were discharged with a final diagnosis suspecting sentinel injury. This analysis compared the rates of conducting diagnostic tests based on the level of the emergency center.

### 2.6. Statistical Analysis

All statistical analyses were performed using R software version 4.3.2 for Windows (R Foundation for Statistical Computing, Vienna, Austria). Frequency analyses were conducted to identify participants’ characteristics. Nominal variables were presented as counts and percentages of the total numbers. Continuous variables without normal distribution were presented as median and interquartile ranges. In addition, all variables were compared using the chi-squared test and Wilcoxon rank sum test at a significance level of *p* < 0.05. The chi-squared test was used to compare the differences in proportions between categorical variables. The Wilcoxon rank sum test was applied to continuous variables that did not meet the assumption of normality. The Shapiro–Wilk test was used to test normality. The Bonferroni correction was applied to adjust the significance level due to the increased risk of a type I error from multiple comparisons across several groups.

## 3. Results

**Figure 1:**
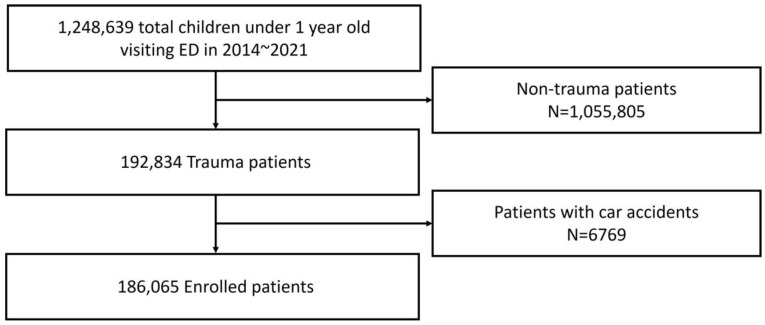
Flow chart.

From 2014 to 2021, the number of pediatric patients under 12 months of age who visited emergency medical centers totaled 1,248,639 nationwide. Of these, 192,834 were trauma pediatric patients. After excluding 6769 pediatric patients involved in car accidents, the final study population comprised 186,065 pediatric patients (Figure 1).

Of the total 186,065 patients, 61,989 (33.3%) were diagnosed with sentinel injuries at discharge from the emergency center, while 124,076 (66.7%) had non-sentinel injuries. The proportion of sentinel injuries was slightly higher in male patients (56.7%). The rate of diagnostic tests conducted for child abuse screening in patients with suspected sentinel injuries was 36.6%, which was lower than 44.0% for those with non-sentinel injuries. Most cases of suspected sentinel injuries (98.4%) were due to accidents, with a small percentage (1.6%) resulting from non-accidental causes. There was no significant difference in the rate of diagnostic tests conducted based on intentionality. Patients with suspected sentinel injuries were less likely to receive specialist consultation compared to those with non-sentinel injuries (39.4% vs. 38.3%). Most patients with suspected sentinel injuries were discharged (98.7%) (Table 1).

**Figure 2:**
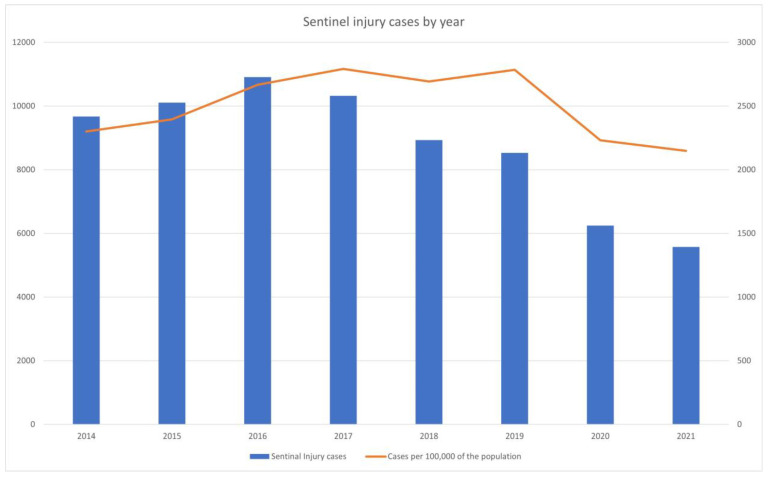
Sentinel injury cases by year. The blue bars represent the actual number of sentinel injury cases for each year from 2014 to 2021. The orange line indicates the cases per 100,000 of the population.

The annual trend of cases with suspected sentinel injuries showed a gradual increase initially, from 9675 cases in 2014, 10,111 in 2015, and 10,908 in 2016 to 10,324 in 2017. However, this trend reversed, with a decrease to 8933 cases in 2018, 8530 in 2019, 6249 in 2020, and finally down to 5575 in 2021. When examining the incidence per 100,000 of the population, there was an increase from 2300 cases in 2014 to 2784 in 2019, but a decrease was observed starting in 2020 (Figure 2).

The rate of diagnostic tests varied according to the type of sentinel injuries. For bruises, diagnostic tests were conducted in 58.2% of cases. However, the rates were lower for burns (3.7%), open wounds (20.5%), and human bites or subconjunctival hemorrhage (13.3%). Looking at individual tests, any X-ray survey was also more frequently performed in cases of bruises (49.1%), but far less so for burns (3.5%), open wounds (17.4%), and human bites or subconjunctival hemorrhage (3.3%). The rate of conducting AST/ALT tests was low across all types of sentinel injuries. Brain imaging was performed in 13.7% of cases with bruises, but was rarely performed for burns (0.0%), open wounds (1.6%), and human bites or subconjunctival hemorrhage (0.0%). Fundus exams were primarily conducted in cases of human bites or subconjunctival hemorrhage (10.8%), and were seldom performed for bruises (0.2%), burns (0.0%), or open wounds (0.7%) (Table 2).

When examining the rate of diagnostic tests conducted for patients with suspected sentinel injuries by the level of emergency centers, it was found that such tests were more frequently performed at regional emergency centers (36.2%) and local emergency centers (38.1%). However, the rate was significantly lower at local emergency institutions, at only 20.0%. This trend was similarly reflected in the rates of X-ray surveys conducted, with the rate in regional emergency centers at 31.5%, local emergency centers at 31.9%, and local emergency institutions at 15.1% (Table 3).

## 4. Discussion

In this study, the number of patients with suspected sentinel injuries fluctuated annually, but overall, it was estimated to be between 2000 and 3000 cases per 100,000 of the population. The continuous increase until 2019 followed by a decrease in 2020 and 2021 is attributed to the decline in emergency center visits due to the COVID-19 pandemic [15,16]. The number of patients with suspected sentinel injuries decreased by approximately 40%, which is lower compared to the 49% reduction in the total number of pediatric patients visiting national emergency centers [17].

Additionally, the rate of specialist consultations was lower for patients with suspected sentinel injuries (28.3% vs. 29.5%), and the rate of conducting diagnostic tests for child abuse screening was also lower (36.6% vs. 44.0%). This could be because most accidental injuries in children are minor and result in emergency visits, and sentinel injuries especially are generally minor injuries [18,19].

In South Korea, the number of child abuse cases per 100,000 of the population has steadily increased from 17.7 cases in 2001 to 502.2 in 2021 [20]. In comparison, data from the United States show that in 2020, there were 4457 child abuse cases per 100,000 of the population, with approximately two deaths due to child abuse per 100,000 of the population [21]. The significantly lower number of child abuse cases in Korea compared to the U.S. is believed to be due to lower public awareness and the consequent underreporting of child abuse in Korea. However, the sharp annual increase in reported child abuse cases suggests an improving awareness of the issue among the public in Korea.

Meanwhile, the number of patients with suspected sentinel injuries identified in this study is much higher than the national statistics for child abuse cases. This suggests that while not all patients with suspected sentinel injuries may be associated with child abuse, national emergency medical data can provide an estimate of the prevalence of sentinel injuries indicative of child abuse surveillance. However, it is not possible to determine what proportion of patients with suspected sentinel injuries in this study experienced child abuse.

Most studies on the correlation between sentinel injuries and child abuse have focused on the prevalence of sentinel injuries among child abuse patients [12,22,23,24]. Sheets et al. demonstrated that among 200 infants who suffered confirmed child abuse, 27.5% had previously experienced a sentinel injury [12]. In a group of 100 infants with moderate concern for abuse, 8% had sustained a sentinel injury, while none of the 101 infants in a group where abuse was ruled out had any sentinel injuries.

However, there appears to be a lack of prospective studies, to the best of the authors’ knowledge, that investigate the proportion of children with sentinel injuries who later become victims of child abuse. Therefore, for this study to be meaningful, further research is needed to determine the proportion of sentinel injury cases that result in child abuse. This would enable the estimation of child abuse cases based on the number of sentinel injuries.

In this study, the rate of conducting diagnostic tests for child abuse screening was found to be lower in cases of sentinel injuries compared to non-sentinel injuries. This lower rate is presumed to be due to the lower severity of sentinel injuries compared to other trauma injuries. There were differences in the rates of conducting diagnostic tests for child abuse screening based on the type of sentinel injury. While diagnostic tests were relatively frequently conducted in cases of bruises, they were less often performed for burns, open wounds, and human bites or subconjunctival hemorrhage. The rate of conducting skeletal surveys was not high for any type of sentinel injury. According to a study by Lindberg et al., the rate of conducting skeletal surveys varied depending on the type of sentinel injury but ranged from 13.1% to 31.9% [22].

While the public awareness of child abuse in South Korea is increasing, awareness among hospital medical staff remains relatively low [25]. The rate of conducting skeletal surveys was too low; when expanded to any X-ray survey, the rate of conducting tests was high for bruises (49.1%) but not for other types of sentinel injuries. This trend is similar to other studies on sentinel injuries and skeletal surveys [19].

This study found variations in the performance of diagnostic tests for suspected sentinel injury patients depending on the level of the emergency center. Notably, the rate of diagnostic tests at regional emergency centers and local emergency centers was relatively high, ranging from 36.2% to 38.1%. In contrast, the rate at local emergency institutions was significantly lower at 20.0%. This finding is consistent with the study by Wood et al., which reported a wide variation in the rate of diagnostic tests for pediatric patients with high-risk injuries for child abuse across 40 major children’s hospitals, ranging from 40% to 90% [24]. Furthermore, according to Trokel et al., the rate at which high-risk injuries like traumatic brain injury and femur fractures were diagnosed as resulting from child abuse was 29% in children’s hospitals, compared to only 13% in general hospitals [26]. Ravichandiran et al. also found that the rate of diagnosing abusive fractures in pediatric emergency departments was seven times higher than in general emergency departments [23]. Based on these findings, it appears that the ability to screen for child abuse varies according to the level or specialty of the hospital. To improve this, we believe that it is necessary to enhance child abuse-related education for all medical professions.

This study has several limitations. First, since it was based on the NEDIS data, the determination of whether an injury was a sentinel injury depended on the diagnostic codes assigned to the patients. Therefore, the precise condition and extent of the trauma in the children were not known. In this study, patients whose primary diagnosis fell within the sentinel injury category were allocated to the sentinel injury group for analysis. However, patients seeking medical attention for minor injuries are more likely to visit primary care facilities than emergency departments. Therefore, future research could improve the accuracy of prevalence estimates by using a database encompassing all healthcare facilities. Additionally, there is a lack of prior research on the actual correlation between child abuse and sentinel injuries as operationally defined by diagnostic codes, making it difficult to estimate the impact of these defined sentinel injuries. Future research is needed to figure out the relationship between sentinel injuries defined by diagnostic codes and actual child abuse. Second, this study, based on NEDIS data, acknowledges that different hospitals might enter different diagnostic codes for the same patient’s condition. For example, a child presenting with a bruise in a specific area could be coded as either a superficial injury or a contusion. To account for this variability, the pre-study Delphi method developed by experts included both superficial injury and contusion in the operational definition of a bruise. However, it should be noted that the severity of a child’s injury can vary, even under the same diagnostic code. Third, the NEDIS database used in this study was not prospectively collected for the purpose of identifying sentinel injuries or child abuse. Therefore, it does not allow for the validation or reliability assessment of the diagnostic results. Additional research is needed, and the authors are currently conducting a study to determine the reliability of this study.

Despite these limitations, this study is meaningful in that it lays the groundwork for further research on the association between sentinel injuries as operationally defined and child abuse. Such research could enable a more accurate national estimate of the scale of child abuse.

## 5. Conclusions

This study presented the national scale and trends of sentinel injuries. A significant number of children visiting emergency centers were found to have sentinel injury codes. Further research is necessary to figure out the association between sentinel injuries as operationally defined and actual cases of child abuse. Additionally, the rate of conducting diagnostic tests for child abuse screening in children with sentinel injury codes was low. There is an urgent need to increase attention to and enhance screening for child abuse among children with sentinel injury codes.

## Figures and Tables

**Table 1:** Demographic differences between sentinel injury and non-sentinel injury patients.

	Non-Sentinel Injury (N = 124,076)	Sentinel Injury (N = 61,989)	*p*
Sex, male	65,939 (53.1%)	35,133 (56.7%)	<0.01
Diagnostic test	54,634 (44.0%)	22,711 (36.6%)	<0.01
Skeletal survey	21 (0.0%)	5 (0.0%)	0.19
Any X-ray survey	45,307 (36.5%)	19,216 (31.0%)	<0.01
Emergency center level			<0.01
Regional emergency center	41,080 (33.1%)	20,511 (33.1%)	
Local emergency center	77,666 (62.6%)	38,466 (62.1%)	
Local emergency institution	5079 (4.1%)	2487 (4.0%)	
Other	251 (0.2%)	525 (0.8%)	
Intention			<0.01
Accidental, unintentional	117,241 (97.1%)	59,278 (98.4%)	
Intentional self-harm	5 (0.0%)	1 (0.0%)	
Violence, assault	156 (0.1%)	76 (0.1%)	
Other specified	1275 (1.1%)	114 (0.2%)	
Unspecified	2099 (1.7%)	784 (1.3%)	
Mechanism of injury			<0.01
Fall	40,430 (33.5%)	20,585 (34.2%)	
Slipped down	7561 (6.3%)	5970 (9.9%)	
Struck by person or object	18,342 (15.2%)	15,927 (26.4%)	
Cut or pierced	5399 (4.5%)	4997 (8.3%)	
Machine	118 (0.1%)	67 (0.1%)	
Fire, flames, or heat	9425 (7.8%)	8343 (13.8%)	
Drowning or nearly drowning	249 (0.2%)	0 (0.0%)	
Poisoning	3426 (2.8%)	14 (0.0%)	
Choking	516 (0.4%)	8 (0.0%)	
Other (sexual assault, electric shock)	31,760 (26.3%)	3181 (5.3%)	
Unknown	3550 (2.9%)	1161 (1.9%)	
Route of arrival			<0.01
Direct visit	117,685 (94.8%)	59,531 (96.0%)	
Transfer from other hospital	6002 (4.8%)	2341 (3.8%)	
Transfer from outpatient facility	334 (0.3%)	103 (0.2%)	
Other/unknown	55 (0.1%)	14 (0.0%)	
Korean Triage and Acuity Scale			<0.01
1	179 (0.2%)	11 (0.0%)	
2	2986 (3.3%)	1321 (2.9%)	
3	12,775 (14.1%)	4919 (10.9%)	
4	63,729 (70.3%)	33,623 (74.3%)	
5	10,903 (12.0%)	5378 (11.9%)	
Other/unknown	32 (0.0%)	10 (0.0%)	
Specialist consultation			<0.01
No	47,513 (38.3%)	24,450 (39.4%)	
Yes	36,612 (29.5%)	17,530 (28.3%)	
Other/unknown	39,951 (32.2%)	20,009 (32.3%)	
Length of stay, minutes	47.0 (25.0–94.0)	44.0 (24.0–86.0)	<0.01
Dispositions			<0.01
Discharge	118,292 (95.6%)	61,117 (98.7%)	
Transfer to other hospital	733 (0.6%)	215 (0.3%)	
Admission	4592 (3.7%)	584 (0.9%)	
Expired	108 (0.1%)	0 (0.0%)	

**Table 2:** Differences in rate of conducting by test type depending on the type of sentinel injury.

	Bruise (N = 33,836)	Burn (N = 11,926)	Open Wound (N = 21,729)	Human Bite or Subconjunctival Hemorrhage (N = 120)	*p*
Diagnostic test	17,898 (58.2%)	345 (3.7%)	4452 (20.5%)	16 (13.3%)	
Any X-ray survey	15,097 (49.1%)	329 (3.5%)	3786 (17.4%)	4 (3.3%)	<0.01
Brain imaging	4200 (13.7%)	4 (0.0%)	351 (1.6%)	0 (0.0%)	<0.01
ALT/AST	52 (0.2%)	35 (0.4%)	27 (0.1%)	0 (0.0%)	<0.01
Fundus exam	71 (0.2%)	0 (0.0%)	162 (0.7%)	13 (10.8%)	<0.01

**Table 3:** Difference in diagnostic tests on patients with sentinel injuries depending on the type of emergency center level.

	Regional Emergency Center (N = 20,511)	Local Emergency Center (N = 38,466)	Local Emergency Institution (N = 2487)	Others (N = 525)	*p*
Diagnostic test	7430 (36.2%)	14,674 (38.1%)	497 (20.0%)	110 (21.0%)	<0.01
Skeletal survey	1 (0.0%)	4 (0.0%)	0 (0.0%)	0 (0.0%)	0.86
Any X-ray survey	6454 (31.5%)	12,281 (31.9%)	376 (15.1%)	105 (20.0%)	<0.01

## Data Availability

The datasets used and/or analyzed during the current study are available from the corresponding authors on reasonable request due to specify the reason for the restriction: legal reasons.
